# Does the application of expandable cages in TLIF provide improved clinical and radiological results compared to static cages? A meta-analysis

**DOI:** 10.3389/fsurg.2022.949938

**Published:** 2022-08-10

**Authors:** Guang-Xun Lin, Jin-Sung Kim, Vit Kotheeranurak, Chien-Min Chen, Bao-Shan Hu, Gang Rui

**Affiliations:** ^1^ Department of Orthopedics, The First Affiliated Hospital of Xiamen University, School of Medicine, Xiamen University, Xiamen, China; ^2^ The Third Clinical Medical College, Fujian Medical University, Fuzhou, China; ^3^ Department of Neurosurgery, Seoul St. Mary's Hospital, College of Medicine, The Catholic University of Korea, Seoul, South Korea; ^4^ Department of Orthopaedics, Faculty of Medicine, Chulalongkorn University, and King Chulalongkorn Memorial Hospital, Bangkok, Thailand; ^5^ Center of Excellence in Biomechanics and Innovative Spine Surgery, Chulalongkorn University, Bangkok, Thailand; ^6^ Division of Neurosurgery, Department of Surgery, Changhua Christian Hospital, Changhua, Taiwan; ^7^ Department of Leisure Industry Management, National Chin-Yi University of Technology, Taichung, Taiwan; ^8^ School of Medicine, Kaohsiung Medical University, Kaohsiung, Taiwan

**Keywords:** expandable cage, static cage, transforaminal lumbar interbody fusion, TLIF, meta-analysis

## Abstract

**Purpose:**

This study aimed to provide a direct meta-analysis of the evidence comparing outcomes between expandable cages and static cages in patients with transforaminal lumbar interbody fusion (TLIF).

**Methods:**

A search of relevant materials from databases was performed from inception to March 7, 2022. Clinical and radiological outcomes were included.

**Results:**

Ten studies (1,440 patients) were included. The anterior disc height and foraminal height for expandable cages were substantially higher than those for static cages at the final follow-up (*P* < 0.0001; *P* = 0.05). In comparison with static cages, although not statistically significant, expandable cages showed beneficial results, including an increase in posterior disc height and segmental lordosis. There were no statistically significant differences in segmental lordosis, lumbar lordosis, pelvic parameters, cage subsidence, or fusion rates (*P* > 0.05). Oswestry disability index scores for expandable cages were substantially lower than those for static cages at the final follow-up (*P* = 0.0007). Interestingly, although the preoperative visual analog scores for back and leg pain were significantly higher in the expandable group than in the static group (*P* < 0.0001; *P* = 0.008), there was no significant difference between the static and expandable groups during the final follow-up (*P* = 0.51; *P* = 0.85).

**Conclusions:**

Expandable cages are associated with improved functional outcomes and restored postoperative disc and foraminal heights in patients with TLIF. In addition, no statistically significant differences were observed in segmental lordosis, lumbar lordosis, pelvic parameters, cage subsidence, or fusion rate.

## Introduction

There are several ways to surgically treat lumbar interbody fusion due to degenerative lumbar diseases; however, each has intrinsic benefits and drawbacks that must be addressed (1–3). The transforaminal lumbar interbody fusion (TLIF) technique improves surgical results while addressing the drawbacks of existing procedures, such as the risk of vascular damage in anterior lumbar interbody fusion and the amount of neural retraction necessary for posterior lumbar interbody fusion (4–6). Static cages have been commonly used in TLIF because they restore disc and foraminal height while potentially enhancing sagittal alignment markers (7, 8). However, the use of static cages in TLIF requires extensive testing, endplate preparation, and overdrawing, which may increase the potential for subsidence and destroy biomechanical stability (9, 10).

Expandable cages were designed to alleviate these difficulties by permitting insertion in a collapsed state and expansion *in situ*, enhancing the ease of insertion, and reducing iatrogenic endplate damage caused by impaction (11, 12). The design of this device may reduce neural retraction, endplate injury, implant subsidence and/or migration, and allow expansion in the interbody space, maximizing the disc space height (13). However, increased expansion may lead to endplate damage and subsidence, and reduced fusion rates (14). Additionally, expandable cages are often more expensive than static cages.

Previous studies have compared the use of expandable cages to static cages in patients undergoing TLIF; however, the included papers were indirect comparative studies (15, 16). Therefore, we performed a comprehensive assessment of the current literature that included direct comparison studies, to evaluate the clinical outcomes and radiographic results of expandable cages versus static cages in patients undergoing TLIF.

## Methods and metairie

### Search strategy

The literature was reviewed and meta-analyzed according to the Preferred Reporting Items for Systematic Reviews and Meta-Analyses (PRISMA) criteria (17). To locate papers involving TLIF employing expandable and static cages, an electronic search of the PubMed, Embase, Scopus, and Web of Science databases was conducted from inception to 7, March 2022. The following keywords were used during the search: “expandable,” “non-expandable,” “static,” “cage,” “spacer,” “transforaminal lumbar interbody fusion,” and “TLIF.” The keywords were concatenated using AND/OR. We also identified relevant publications from the literature to aid in our search. For additional research, the references of selected papers were evaluated.

### Study selection

The following criteria were used to select studies for inclusion in the meta-analysis: (1) described at least one of the primary outcomes of interest in patients who underwent TLIF with static or expandable cage implantation, (2) published in the English language, and (3) reported follow-up outcomes at a minimum of 6 months. Clinical trials, both non-randomized and randomized, as well as comparative observational studies and case series, were included. Abstracts, case reports, review articles, and cadaveric or biomechanical studies were not included. Two researchers independently examined the titles and abstracts of the search results. The relevance of the selected papers was then assessed. Any issues were resolved through a discussion with a third party.

### Data extraction

Clinical and radiological results were the major outcomes of this study. Clinical outcomes included preoperative and postoperative visual analog scale (VAS) scores for back and leg pain, as well as Oswestry Disability Index (ODI) scores. Radiological outcomes comprised preoperative and postoperative data, including anterior disc height (ADH), posterior disc height (PDH), foraminal height (FH), segmental lordosis (SL), lumbar lordosis (LL), pelvic tilt (PT), sacral slope (SS), pelvic incidence – LL (PI-LL) mismatch, cage subsidence, and fusion rates (8).

### Quality evaluation

The Newcastle–Ottawa Scale was used to assess the quality of the non-randomized trials (1). Each study was evaluated according to selection, comparability, and exposure/outcome. Using these criteria, we considered papers that obtained more than five “stars” in our review.

### Statistical analysis

RevMan version 5.4 (Cochrane Collaboration, Oxford, UK) was used to analyze the data. The mean differences and 95% confidence intervals (CI) for continuous data are provided. Dichotomous factors were analyzed in comparative studies using odds ratios (OR) or risk ratios. Continuous variables were evaluated using the weighted mean differences (WMD) or standard mean differences. The x^2^ and I^2^ tests were used to investigate heterogeneity, with *P* > 0.1 or I^2^ < 50% being homogenous across trials, and a fixed-effects model was used. A random-effects model was used if I^2^ was >50%. To evaluate statistical significance, a *P*-value of 0.05 was employed. Forest plots were created to graphically represent the findings of several studies and the aggregated effect estimates.

## Results

### Study selection and quality evaluation

**Figure 1:**
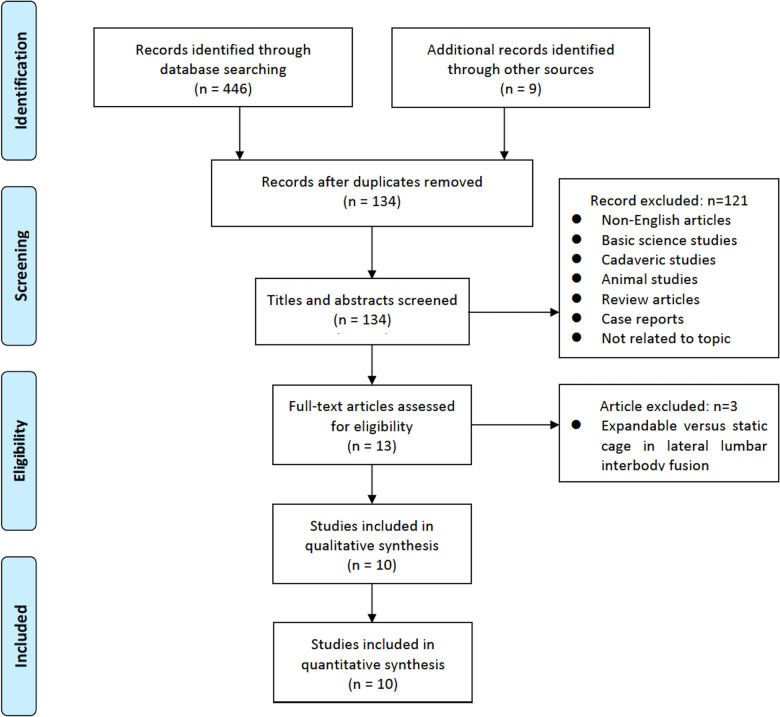
Study selection flow diagram for the meta-analysis.

A total of 134 studies were initially identified. Following a review of the titles and abstracts, 121 articles were excluded. The remaining 13 papers were thoroughly reviewed, and among them, 10 papers satisfied the inclusion criteria and were included in the analysis. The full search procedure is shown in the PRISMA flowchart (Figure 1).

The 10 selected studies included a total of 1,440 patients, with 661 and 779 individuals recruited in the expandable and static groups, respectively. Nine of the 10 papers are from the United States, and one is from Taiwan region. The L4–L5 section was most often operated upon. The average ages of the expandable and static groups were 63.13 and 56.45 years, respectively. Demographic information is summarized in Table 1.

**Table 1:** Characteristics of the included studies.

Study	Study design	Country/ Region	No. of cases	Diagnosis	Operative level	Age (years)	Sex (M/F)	Follow-up (months)
Canseco 2021 (18)	Retrospective	USA	Expandable (103)	Spondylolisthesis (40); Stenosis (57); Disc herniation (5); Deformity (1)	L1-2 (3); L2-3 (8); L3-4 (12); L4-5 (59); L5-S1 (21)	63.9 ± 9.08	49/54	12
			Static (137)	Spondylolisthesis (80); Stenosis (33); Disc herniation (17); Deformity (7)	L2-3 (1); L3-4 (11); L4-5 (97); L5-S1 (28)	61.6 ± 11.4	61/76	
Chang 2021 (8)	Retrospective	Taiwan	Expandable (62) Static (148)	Isthmic spondylolisthesis (6); Recurrent stenosis (1); Degenerative spondylolisthesis w/ stenosis (46); Severe spondylosis w/ intractable low-back pain or leg pain (9)	L2-3 (1); L3-4 (8); L4-5 (41); L5-S1 (5)	62.8 ± 14.1	31/31	27.6 ± 14.1
				Isthmic spondylolisthesis (14); Recurrent stenosis (16); Degenerative spondylolisthesis w/ stenosis (29); Severe spondylosis w/ intractable low-back pain or leg pain (89)	L2-3 (2); L3-4 (20); L4-5 (93); L5-S1 (33)	60.3 ± 11.5	51/97	42.9 ± 29.4
Gelfand 2020 (9)	Retrospective	USA	Expandable (67)	Spondylolisthesis	NR	61.9	30/37	NR
			Static (47)			54.9	19/28	
Hawasli 2017 (19)	Retrospective	USA	Expandable (28)	DDD and lumbar spondylosis with radiculopathy with or without Grade I to II spondylolisthesis and the absence of previous surgical instrumentation	L2-3 (4); L3-4 (4); L4-5 (20); L5-S1 (1)	63.9 ± 9.9	15/13	7.1 ± 4.2
			Static (16)		L3-4 (3); L4-5 (14); L5-S1 (2)	57.7 ± 8.9	10/6	14.6 ± 7.1
Khechen 2020 (10)	Retrospective	USA	Expandable (30)	Single-level degenerative pathology	L3-4 (2); L4-5 (19); L5-S1 (9)	52.2 ± 12.1	23/7	6
			Static (30)		L4-5 (19); L5-S1 (11)	53.5 ± 11.7	19/11	
Kremer 2019 (12)	Retrospective	USA	Expandable (51)	Degenerative spondylolisthesis w/o HNP (25); Stenosis w/o HNP (11); DDD w/o radiculopathy (14); Adult degenerative scoliosis with stenosis (1)	NR	62.8 ± 13.5	NR	43.0 ± 4.2
			Static (48)	Degenerative spondylolisthesis w/o HNP (13); Stenosis w/o HNP (21); DDD w/o radiculopathy (12); Radiculopathy (2)		58.3 ± 13.7		67.1 ± 16.3
Russo 2021 (20)	Retrospective	USA	Expandable (27)	DDD at one level from L2 to S1 with or without Grade 1 spondylolisthesis	L3-4 (3); L4-5 (10); L5-S1 (14)	55.7 ± 9.5	15/12	9.1
			Static (21)		L4-5 (10); L5-S1(10); L6-S1 (1)	52.1 ± 11.9	9/12	16.0
Vaishnav 2020 (14)	Retrospective	USA	Expandable (60)	Degenerative conditions of the spine	Single-level	64	26/34	NR
			Static (111)			58	62/49	
Woodward 2022 (7)	Retrospective	USA	Expandable (60)	Grade I spondylolisthesis and/or DDD	NR	63.5	29/31	12
			Static (60)			59.3 ± 9.86	19/41	
Yee 2017 (21)	Retrospective	USA	Expandable (41)	Recurrent HNP (7); Stenosis) (9); Scoliosis (3); Spondylolisthesis (22)	L3-4 (4); L4-5 (15); L5-S1 (22)	54.5 ± 13.8	21/20	12
			Static (48)	Recurrent HNP (5); Stenosis) (3); Scoliosis (2); Spondylolisthesis (38)	L3-4 (1); L4-5 (29); L5-S1 (18)	58.3 ± 13.4	20/28	

HNP, herniated nucleus pulposus; DDD, degenerative disc disease; NR, not reported; w/o, with or without.

According to the Newcastle–Ottawa Scale evaluation, all 10 studies had a retrospective comparative cohort design and were of moderate-to-high quality (Table 2).

**Table 2:** Quality assessment of the included studies.

Studies	Selection				Comparability	Exposure			Total scores (of 9)
	Is the case definition adequate?	Representativeness of the cases	Selection of Controls	Definition of Controls	Comparability of cases and controls on the basis of the design or analysis	Ascertainment of exposure	Same method of ascertainment for cases and controls	Non-Response rate	
Canseco 2021 (18)	☆		☆	☆	☆☆	☆	☆		7☆
Chang 2021 (8)	☆		☆	☆	☆☆	☆	☆		7☆
Gelfand 2020 (9)	☆		☆	☆	☆☆	☆	☆		7☆
Hawasli 2017 (19)	☆	☆	☆	☆	☆☆	☆	☆		8☆
Khechen 2020 (10)	☆	** **	☆	☆	☆☆	☆	☆	** **	7☆
Kremer 2019 (12)	☆	** **	☆	☆	☆☆	☆	☆	** **	7☆
Russo 2021 (20)	☆	** **	☆	☆	☆☆	☆	☆	** **	7☆
Vaishnav 2020 (14)	☆	** **	☆	☆	☆☆	☆	☆	** **	7☆
Woodward 2022 (7)	☆	☆	☆	☆	☆☆	☆	☆	** **	8☆
Yee 2017 (21)	☆	** **	☆	☆	☆☆	☆	☆	** **	7☆

### Radiological parameters

**Figure 2:**
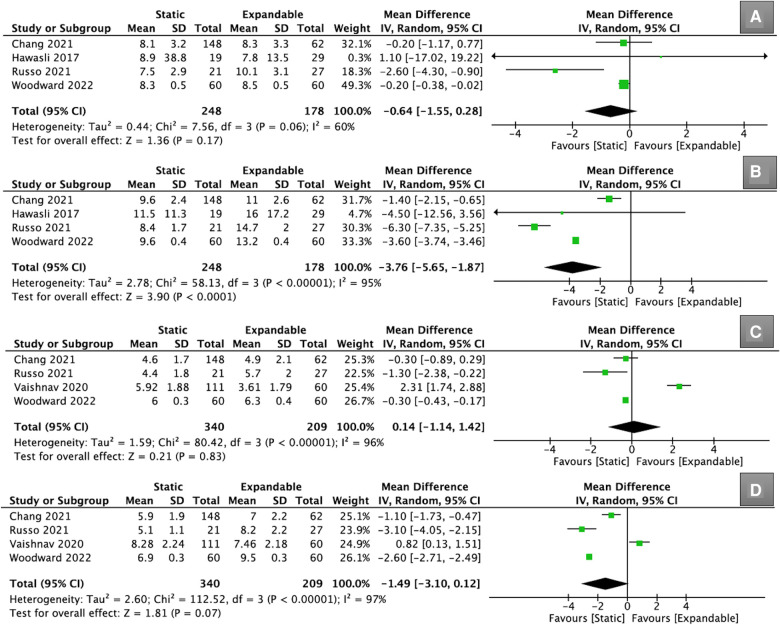
Forest plots for comparison of ADH at preoperative (**A**) and final follow-up (**B**) between the static and expandable groups. Forest plots for comparison of PDH at preoperative (**C**) and final follow-up (**D**) between the static and expandable groups. ADH: anterior disc height; PDH: posterior disc height.

Four studies (*n* = 426) reported data on ADH. Preoperatively, there was no significant difference in ADH between the static and expandable groups (WMD, −0.64; 95% CI, −1.55, 0.28; I^2^ = 60%; *P* = 0.17; Figure 2A). It is worth noting that the ADH for expandable cages was substantially higher than that for static cages at the final follow-up (WMD, −3.76; 95% CI, −5.65, −1.87; I^2^ = 95%; *P* < 0.0001; Figure 2B). This indicated that the mean increase in ADH was greater in expandable cages than in static cages. The mean ADH in the static group increased from 8.2 mm preoperatively to 9.8 mm at the final follow-up, whereas the mean ADH in the expandable group increased from 8.7 mm preoperatively to 13.7 mm at the final follow-up.

Four other studies (*n* = 549) reported data on PDH. However, there was no significant difference in PDH between the static and expandable groups preoperatively (WMD, 0.14; 95% CI, −1.14, 1.42; I^2^ = 96%; *P* = 0.83; Figure 2C) and at the final follow-up (WMD, −1.49; 95% CI, −3.10, 0.12; I^2^ = 97%; *P* = 0.07; Figure 2D). The mean PDH in the static group increased from 5.2 mm preoperatively to 6.5 mm at the final follow-up, whereas the mean PDH in the expandable group increased from 5.1 mm preoperatively to 8.0 mm at the final follow-up. Although there was no statistical difference in PDH between the two groups at the final follow-up, it should be noted that the mean change in PDH was greater in the expandable group.

**Figure 3:**
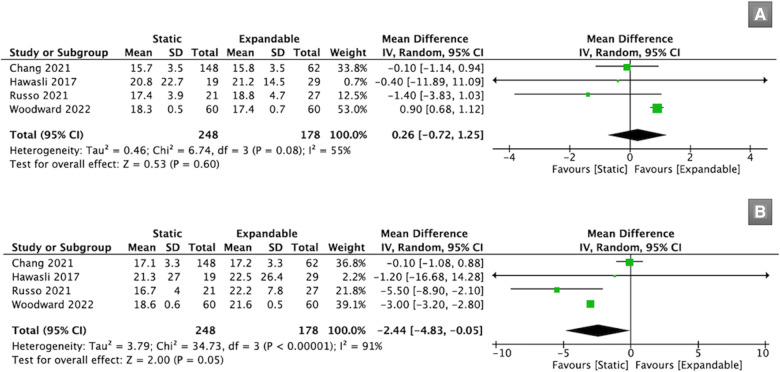
Forest plots for comparison of FH at preoperative (**A**) and final follow-up (**B**) between the static and expandable groups. FH: foraminal height.

Four studies (*n* = 426) reported data about the FH. Preoperatively, there was no significant difference in FH between the static and expandable groups (WMD, 0.26; 95% CI, −0.72, 1.25; I^2^ = 55%; *P* = 0.60; Figure 3A). However, the FH for expandable cages was substantially larger than that for static cages at the final follow-up (WMD, −2.44; 95% CI, −4.83, −0.05; I^2^ = 91%; *P* = 0.05; Figure 3B). The mean FH in the static group increased from 18.1 mm preoperatively to 18.4 mm at the final follow-up, whereas the mean FH in the expandable group increased from 18.5 mm preoperatively to 20.9 mm at the final follow-up.

**Figure 4:**
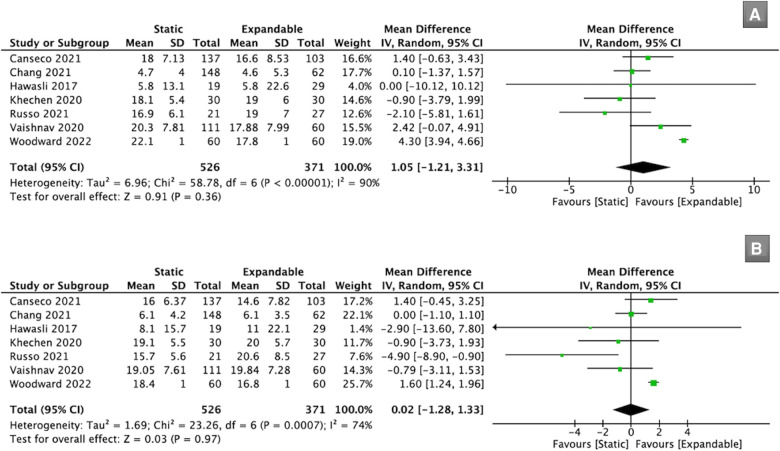
Forest plots for comparison of SL at preoperative (**A**) and final follow-up (**B**) between the static and expandable groups. SL: segmental lordosis.

Seven studies (*n* = 897) reported SL data. There was no significant difference in SL between the static and expandable groups preoperatively (WMD, 1.05; 95% CI, −1.21, 3.31; I^2^ = 90%; *P* = 0.36; Figure 4A) and at the final follow-up (WMD, 0.02; 95% CI, −1.28, 1.33; I^2^ = 74%; *P* = 0.97; Figure 4B). The mean SL decreased from 15.13° preoperatively to 14.64° at the final follow-up in the static group. However, in the expandable group, the mean SL increased from 14.38° preoperatively to 15.56° at the final follow-up. This may indirectly prove that SL can be improved using expandable cages compared to that using static cages.

**Figure 5:**
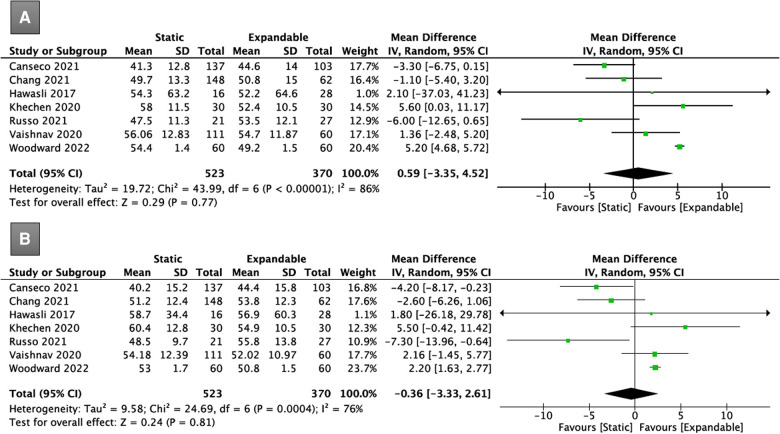
Forest plots for comparison of LL at preoperative (**A**) and final follow-up (**B**) between the static and expandable groups. LL: lumbar lordosis.

Seven studies (*n* = 893) reported LL data. There was no significant difference in LL between the static and expandable groups preoperatively (WMD, 0.59; 95% CI, −3.35, 4.52; I^2^ = 86%; *P* = 0.77; Figure 5A) and at the final follow-up (WMD, −0.36; 95% CI, −3.33, 2.61; I^2^ = 76%; *P* = 0.81; Figure 5B). The mean LL in the static group increased from 51.6° preoperatively to 52.3° at the final follow-up, whereas the mean LL in the expandable group increased from 51.1° preoperatively to 52.7° at the final follow-up.

**Figure 6:**
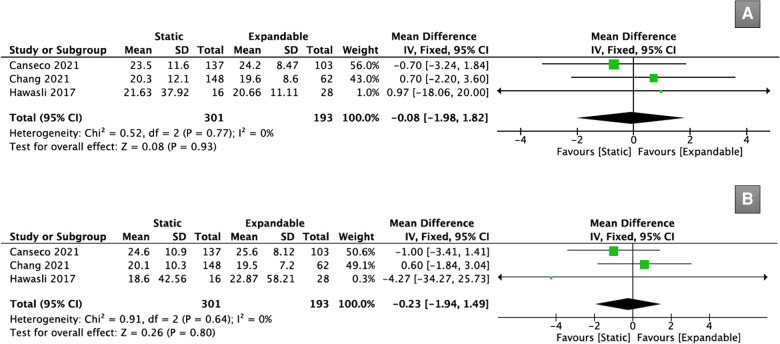
Forest plots for comparison of PT at preoperative (**A**) and final follow-up (**B**) between the static and expandable groups. PT: pelvic tilt.

Three studies (*n* = 494) reported PT data. There was no significant difference in PT between the static and expandable groups preoperatively (WMD, −0.08; 95% CI, −1.98, 1.82; I^2^ = 0%; *P* = 0.93; Figure 6A) and at the final follow-up (WMD, −0.23; 95% CI, −1.94, 1.49; I^2^ = 0%; *P* = 0.80; Figure 6B). The mean PT in the static group decreased from 21.8° preoperatively to 21.1° at the final follow-up, whereas the mean PT in the expandable group increased from 21.5° preoperatively to 22.7° at the final follow-up.

**Figure 7:**
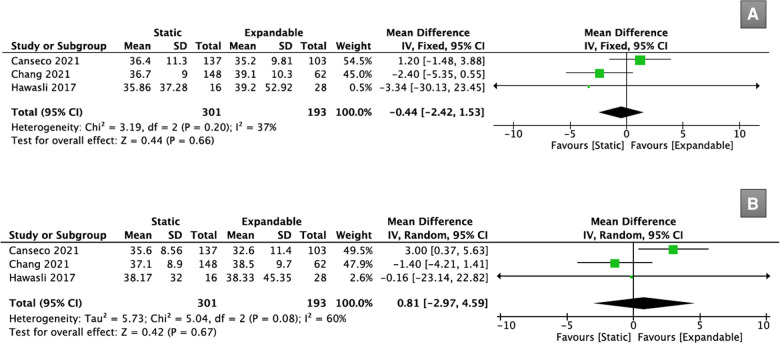
Forest plots for comparison of SS at preoperative (**A**) and final follow-up (**B**) between the static and expandable groups. SS: sacral slope.

Three studies (*n* = 494) reported SS data. There was no significant difference in SS between the static and expandable groups preoperatively (WMD, −0.44; 95% CI, −2.42, 1.53; I^2^ = 37%; *P* = 0.66; Figure 7A) and at the final follow-up (WMD, 0.81; 95% CI, −2.97, 4.59; I^2^ = 60%; *P* = 0.67; Figure 7B). The mean SS in the static group increased from 36.3° preoperatively to 37.0° at the final follow-up, whereas the mean SS in the expandable group decreased from 37.8° preoperatively to 36.5° at the final follow-up.

**Figure 8:**
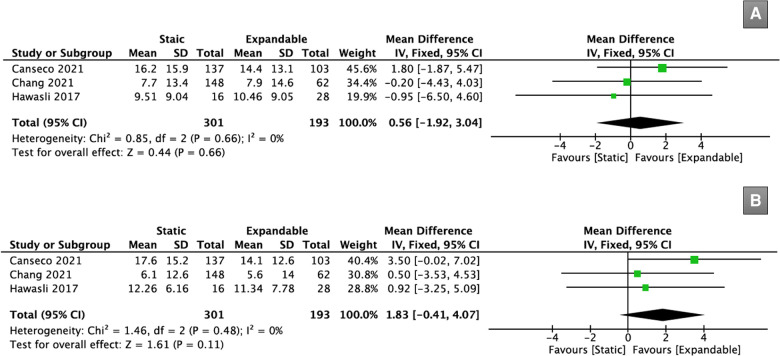
Forest plots for comparison of PI-LL mismatch at preoperative (**A**) and final follow-up (**B**) between the static and expandable groups.

Three studies (*n* = 494) reported data on PI-LL mismatch. There was no significant difference in the PI-LL mismatch between the static and expandable groups preoperatively (WMD, 0.56; 95% CI, −1.92, 3.04; I^2^ = 0%; *P* = 0.66; Figure 8A) or postoperatively (WMD, 1.83; 95% CI, −0.41, 4.07; I^2^ = 0%; *P* = 0.11; Figure 8B). The mean PI-LL mismatch in the static group increased from 11.1° preoperatively to 11.9° at the final follow-up, whereas the mean PI-LL mismatch in the expandable group decreased from 10.9° preoperatively to 10.3° at the final follow-up.

**Figure 9:**
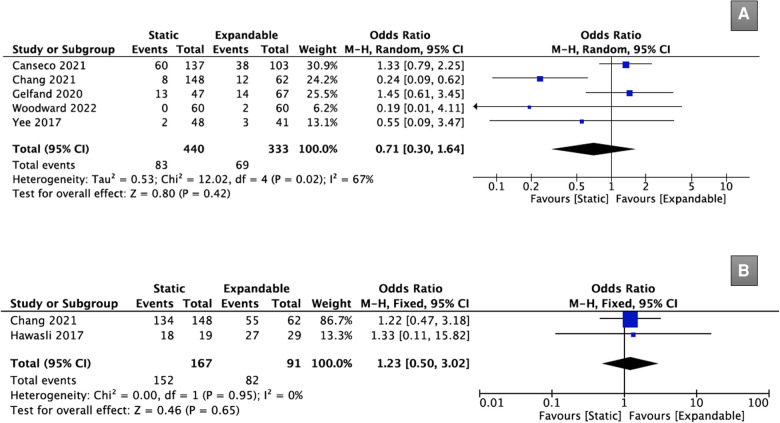
Forest plots comparing case subsidence (**A**), and fusion rate (**B**) between the static and expandable groups. PI-LL: pelvic incidence – lumbar lordosis.

Five studies (*n* = 773) reported cage subsidence data. There was no significant difference in cage subsidence between the static (18.9%) and expandable (20.7%) groups after TLIF (OR, 0.71; 95% CI, 0.30, 1.64; I^2^ = 67%; *P* = 0.42; Figure 9A).

Only two studies (*n* = 258) provided data on fusion rates after TLIF. There was no significant difference in the fusion rates between the static (91.0%) and expandable (90.1%) groups after TLIF (OR, 1.23; 95% CI, 0.50, 3.02; I^2^ = 0%; *P* = 0.65; Figure 9B).

### Clinical outcomes

**Figure 10:**
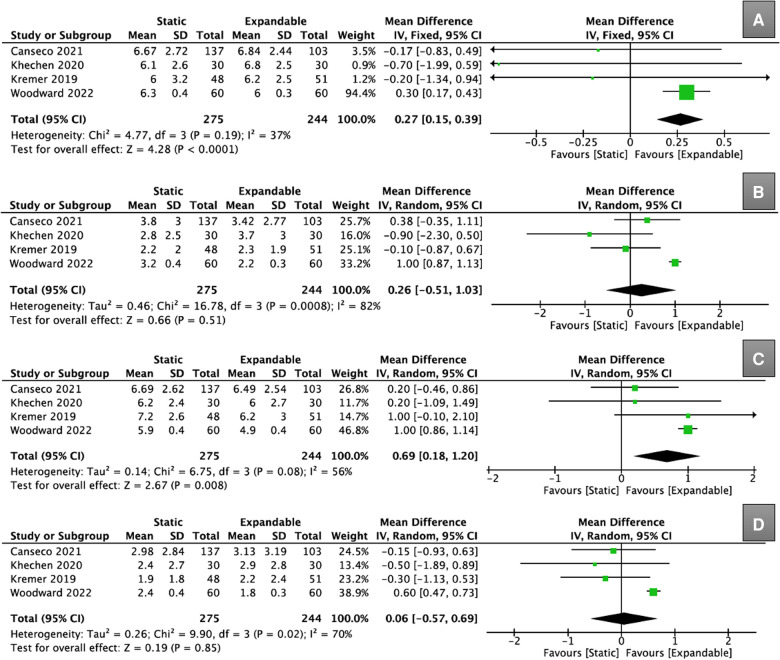
Forest plots for comparison of VAS for back at preoperative (**A**) and final follow-up (**B**) between the static and expandable groups. Forest plots for comparison of VAS for leg at preoperative (**C**) and final follow-up (**D**) between the static and expandable groups.VAS: visual analog scale.

Four studies (*n* = 519) provided data on VAS scores for back pain. Although the preoperative VAS scores for back pain were significantly higher in the expandable group than in the static group (WMD, 0.27; 95% CI, 0.15, 0.39; I^2^ = 37%; *P* < 0.0001; Figure 10A), there was no significant difference between the static and expandable groups at the final follow-up (WMD, 0.26; 95% CI, −0.51, 1.03; I^2^ = 82%; *P* = 0.51; Figure 10B). The mean VAS score for back pain in the static group decreased from 6.3 preoperatively to 3.0 at the final follow-up, whereas the mean VAS score for back pain in the expandable group decreased from 6.5 preoperatively to 2.9 at the final follow-up.

Four studies (*n* = 519) provided data on VAS scores for leg pain. Although the preoperative VAS score for leg pain was significantly higher in the expandable group than in the static group (WMD, 0.69; 95% CI, 0.18, 1.20; I^2^ = 56%; *P* = 0.008; Figure 10C), there was no significant difference between the static and expandable groups at the final follow-up (WMD, 0.06; 95% CI, −0.57, 0.69; I^2^ = 70%; *P* = 0.85; Figure 10D). The mean VAS score for leg pain in the static group decreased from 6.5 preoperatively to 2.4 at the final follow-up, whereas the mean VAS score for leg pain in the expandable group decreased from 5.9 preoperatively to 2.5 at the final follow-up.

**Figure 11:**
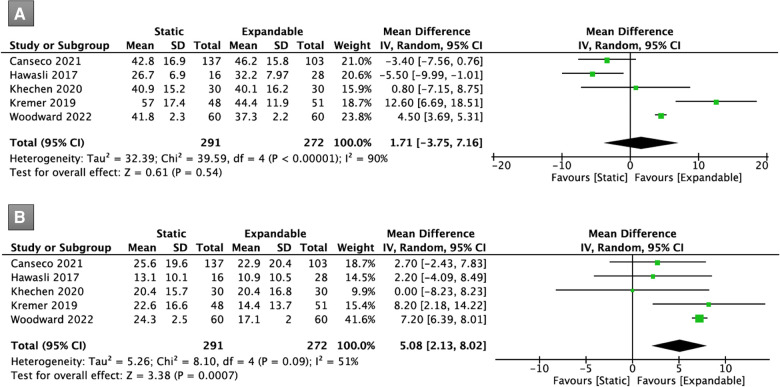
Forest plots for comparison of ODI at preoperative (**A**) and final follow-up (**B**) between the static and expandable groups. ODI: Oswestry Disability Index.

Five studies (*n* = 563) reported data on ODI scores. Preoperatively, there was no significant difference in the ODI scores between the static and expandable groups (WMD, 1.71; 95% CI, −3.75, 7.16; I^2^ = 90%; *P* = 0.54; Figure 11A). It is worth mentioning that the ODI scores for expandable cages were found to be substantially lower than for static cages at the final follow-up (WMD, 5.08; 95% CI, 2.13, 8.02; I^2^ = 51%; *P* = 0.0007; Figure 11B). The mean ODI score in the static group decreased from 41.8 preoperatively to 21.2 at the final follow-up, whereas the mean ODI score in the expandable group decreased from 40.0 preoperatively to 17.1 at the fina follow-up.

## Discussion

Recent advances in interbody cage design have led to substantial progress in disc distraction, sagittal alignment correction, cage subsidence or migration, and fusion rate (20, 21). Among these, expandable cages have emerged as attractive alternatives to standard static cages for lumbar interbody fusion surgery. Implantation of an expandable cage provides the benefit of reducing nerve root discomfort and endplate compression and enhancing the recovery of spinal curvature (19). Previous systematic studies have compared the use of extendable cages to static cages in patients undergoing TLIF; however, none of the included studies were direct comparative studies between the two groups. Therefore, we collected direct comparison papers for a meta-analysis to evaluate the clinical and radiological outcomes of patients undergoing TLIF using expandable cages versus static cages. This study showed that employing either expandable or static interbody spacers following TLIF results in positive radiographic and functional outcomes.

According to our findings, compared to static cages, the use of expandable cages in TLIF was linked to significant restoration of ADH and FH. Although the comparison with static cages failed to reach statistical significance, expandable cages showed positive results, including an increase in the PDH. Expanding technology enables the insertion of a larger cage and promotes disc distraction, resulting in an increase in PDH and FH (14, 18). In addition, the use of an expandable cage allows it to be placed anterior to the disc space, as the device articulates to the anterior annulus of the vertebral body to increase biomechanical stability, which is the strongest part of the disc space, resulting in improvement in the superior disc height and FH (7, 19). It is worth noting that our analysis shows that SL decreased in the static group and increased in the expandable group. Although this change does not reach a statistically significant difference, it can indirectly indicate that using an expandable cage to restore SL is better than using the static cage. The use of expandable cages has been proposed to be advantageous not only for LL but also for restoring disc height and improving SL. However, our analysis found that the use of expandable cages in TLIF may not result in a significantly improved correction of LL compared to static cages. These findings suggest that the expansion of disc height and SL provided by an expandable cage does not adequately correct LL.

Some researchers have suggested that failing to restore anterior lumbar convexity has a detrimental impact on patient outcomes, such as unequal load distribution in the posterior vertebral body, irritation of spinal tissues, lower back discomfort, and postural instability (22, 23). In addition, improvements in postoperative pain and functional outcomes have been linked to the restoration of spinal sagittal alignment (19). However, in terms of spinal sagittal alignment (SL, LL, PT, SS, and PI-LL mismatch), our findings showed no significant difference in postoperative changes between the two groups with respect to any radiological parameter. The improvement in functional outcomes was a noteworthy result of this study, regardless of radiographic data. The VAS scores for back and leg pain were significantly reduced regardless of the type of cage used, but no significant difference was observed between the two types of cages. Interestingly, the functional outcomes (ODI scores) for the expandable cage group were significantly better than those of the static cage group.

Cage subsidence in interbody fusion surgery is a major issue because it may result in lordosis loss and adjacent segment degeneration, necessitating revision surgery (24). Theoretically, the installation of an expandable cage results in less damage to the endplate. This is consistent with our own experience of utilizing these cages during surgery. However, no significant difference was observed in cage subsidence between the static (18.9%) and expandable (20.7%) groups after TLIF.

The fusion rates were similar between the two groups (90.1% for the expandable cage group vs. 91.0% for the static cage group; *P* = 0.62). However, it is vital to highlight that the majority of research differs in some key factors that could influence this finding. The type of bone graft used is known to affect the outcome of fusion, but most papers that were included in this analysis did not mention the specific type. Moreover, depending on the type and design of the expandable cage, the ability to insert sufficient graft material within the cage varies and may result in discrepancies in fusion rates. In addition, only two studies in this meta-analysis provided data on the fusion rate (8, 19); therefore, the evidence of the results needs to be improved.

This is the first systematic review and meta-analysis study that directly compared expandable cages with static cages and gives many meaningful results. However, there were inevitable limitations to this study. First, the level of evidence was low because of the retrospective nature of all included investigations. Second, data on the outcomes and study cohort heterogeneity were lacking. Third, no subgroup analysis of minimally invasive TLIF versus open TLIF was performed. Finally, long-term results were not obtained in this study. Owing to the aforementioned considerations, high-quality research is required to prove the relative advantages of expandable cages versus static cages in TLIF.

## Conclusions

Expandable cages are positively associated with restored postoperative disc and foraminal heights and improved functional outcomes in patients with TLIF. In addition, there were no statistically significant differences in spinal sagittal alignment (SL, LL), pelvic parameters (PT, SS, and PI-LL mismatch), cage subsidence, or fusion rates.

## Data Availability

The original contributions presented in the study are included in the article/Suplementary Material, further inquiries can be directed to the corresponding author/s.
